# Brain haemosiderin in older people: pathological evidence for an ischaemic origin of magnetic resonance imaging (MRI) microbleeds

**DOI:** 10.1111/nan.12062

**Published:** 2014-03-13

**Authors:** B M Janaway, J E Simpson, N Hoggard, J R Highley, G Forster, D Drew, O H Gebril, F E Matthews, C Brayne, S B Wharton, P G Ince

**Affiliations:** * Sheffield Institute for Translational Neuroscience, University of SheffieldSheffield, UK; † Academic Unit of Radiology, University of SheffieldSheffield, UK; § MRC Biostatistics Unit, University of CambridgeCambridge, UK; ¶ Institue of Public Health, University of CambridgeCambridge, UK; ‡ Medical Research Division, National Research CentreCairo, Egypt

**Keywords:** haemorrhage, haemosiderin, ischaemia, microbleeds, small vessel disease, stroke

## Abstract

**Introduction:**

Magnetic resonance imaging (MRI) cerebral microbleeds (CMB) arise from ferromagnetic haemosiderin iron assumed to derive from extravasation of erythrocytes. Light microscopy of ageing brain frequently reveals foci of haemosiderin from single crystalloids to larger, predominantly perivascular, aggregates. The pathological and radiological relationship between these findings is not resolved.

**Methods:**

Haemosiderin deposition and vascular pathology in the putamen were quantified in 200 brains donated to the population-representative Medical Research Council Cognitive Function and Ageing Study. Molecular markers of gliosis and tissue integrity were assessed by immunohistochemistry in brains with highest (*n* = 20) and lowest (*n* = 20) levels of putamen haemosiderin. The association between haemosiderin counts and degenerative and vascular brain pathology, clinical data, and the haemochromatosis (*HFE*) gene H63D genotype were analysed. The frequency of MRI CMB in 10 cases with highest and lowest burden of putamen haemosiderin, was compared using *post mortem* 3T MRI.

**Results:**

Greater putamen haemosiderin was significantly associated with putaminal indices of small vessel ischaemia (microinfarcts, *P* < 0.05; arteriolosclerosis, *P* < 0.05; perivascular attenuation, *P* < 0.001) and with lacunes in any brain region (*P* < 0.023) but not large vessel disease, or whole brain measures of neurodegenerative pathology. Higher levels of putamen haemosiderin correlated with more CMB (*P* < 0.003).

**Conclusions:**

The MRI-CMB concept should take account of brain iron homeostasis, and small vessel ischaemic change in later life, rather than only as a marker for minor episodes of cerebrovascular extravasation. These data are of clinical relevance, suggesting that basal ganglia MRI microbleeds may be a surrogate for ischaemic small vessel disease rather than exclusively a haemorrhagic diathesis.

## Introduction

Cerebral microbleeds (CMB) appear as small (generally <5-mm diameter), magnetic resonance imaging (MRI) signal voids best demonstrated in susceptibility-weighted and gradient echo density scanning sequences. They are often assumed to reflect microscopic accumulation of haemosiderin deposits 1,2. MRI investigations have indicated that CMB are prevalent in approximately 5–6% of the normal population. The prevalence increases in normal ageing where the majority of CMB occur in deep brain structures, including the putamen 3,4, and in patients with hypertension, cerebral ischaemia, intracerebral haemorrhage and stroke 5. An assumption appears to have arisen, on the basis that the CMB imaging artefact is caused by paramagnetic properties of haemosiderin iron, that they arise from processing of extravasated erythrocyte haemoglobin. Combined radiological and histological study in *post mortem* tissue has demonstrated a strong correlation between microhaemorrhages and MRI CMB in the context of cerebral amyloid angiopathy (CAA) 6. The histopathology of CAA is frequently associated with evidence of microhaemorrhages and the clinical manifestations include lobar haemorrhages 7. In circumstances other than CAA it has been suggested that age-related changes in the structure of the blood–brain barrier may result in opening of endothelial junctions thereby allowing egress of red blood cells, resulting in CMB 3,8,9. However CMB are also well described in the context of CADASIL, a brain disorder in which characteristic vascular sclerosis is not associated with pathological evidence of acute microhaemorrhage and in which clinical intracerebral haemorrhage is very rare 10,11. Taken with the association of CMB with cerebral infarction, such findings raise the possibility that haemosiderin deposition in the ageing brain may accumulate from sources other than extravasated erythrocytes. For the purposes of this report the term CMB will be exclusively used in the context of the MR paramagnetic artefact. In contrast the histological appearances are described as focal haemosiderin throughout.

Uptake of iron into the brain is unidirectional, complex, and facilitated by receptor-mediated endocytosis of iron bound to transferrin 12. Iron accumulation with age in post mitotic tissues, especially the brain, is well documented and thought to arise from the absence of a functional export pathway 13,14. Oligodendroglia are the major reservoir of brain iron storage within ferritin, and iron content is highest in basal ganglia 13. The term ferritin refers to fully assembled iron-containing shells. The apoprotein units that comprise the shell are composed of a mixture of ferritin light and ferritin heavy derived from two different genes. In the present study we have sought evidence to support an alternative hypothesis for the origin of haemosiderin foci (and by implication the source of MRI CMB) based on increasing saturation of iron storage in older brains as a consequence of unidirectional iron uptake. An important consideration is the nature and origin of haemosiderin. It does not represent a specific molecular intermediate of haemoglobin degradation. Rather it is formed within secondary lysosomes as a complex of ferritin, iron and proteins (including membrane proteins) produced in any circumstances of iron overload of macrophages and other cell types 15. Haemosiderin formation is most marked in pathological disorders associated with iron overload rather than as a biomarker of previous episodes of bleeding 16. When ischaemia due to small vessel disease (SVD) damages brain tissue, the release of stored iron from oligodendroglia and other cells, and of the iron incorporated into haem-containing proteins, may exceed the ability of the surrounding tissue to process it into new ferritin/iron stores. A local excess of iron could therefore be processed by macrophages to haemosiderin and transported to a perivascular location to assist macrophage-mediated clearance. This hypothesis can be addressed in part through certain predictions:

- Focal haemosiderin deposition will be significantly associated with local indices of ischaemic SVD in comparison with large vessel disease and vascular pathology in other brain regions.
- Focal haemosiderin deposition will be more prominent in people whose brain is predisposed to increased iron uptake for example associated with pathogenic *HFE* gene mutations.
- People with a higher burden of focal haemosiderin deposits in one brain region will have more CMB in other brain areas based on the usual widespread impact of SVD.

The aim of the present study was to address these predictions histologically by quantifying putamen haemosiderin deposition in an unselected, population-based cohort of elderly individuals from the Medical Research Council Cognitive Function and Ageing Study (MRC CFAS) 17. We assessed the relationship between haemosiderin deposition and a variety of measures, including local vascular pathology, global brain pathology scores, dementia status, clinical risk factors for vascular disease, and the *HFE* H63D genotype. The relationship between histologically identified haemosiderin and CMB MRI voids was determined in a subgroup of cases. Such data can only address the specific hypothesis that brain haemosiderin deposits are related to the severity of local vascular pathology. They do not address the underlying hypothesis that the source of this haemosiderin is predominantly derived from oligodendrocyte ferritin and glioneuronal haem-containing proteins rather than from erythrocyte breakdown.

## Materials and methods

### Human central nervous system (CNS) material

Human CNS tissue from 200 brain donors was obtained from MRC CFAS autopsy cohort. CFAS is a longitudinal, prospective population-representative study in which brain donor recruitment was solely based on age (over 65 years) 18 and was unrelated to dementia or other clinical data. The donated brains were pathologically assessed by neuropathologists following the Consortium to Establish a Registry of Alzheimer's Disease (CERAD) protocol 19 and Braak staging 20 in addition to assessments of vascular pathology, including arteriosclerosis, atheroma, SVD, microinfarction, lacunes and parenchymal integrity. All cases were previously screened for the *HFE* H63D polymorphism 21. Multi-Centre Research Ethical Committee approval was given for all procedures.

### Focal haemosiderin deposition, local vascular pathology, global vascular and degenerative pathology, clinical data

**Figure 1:**
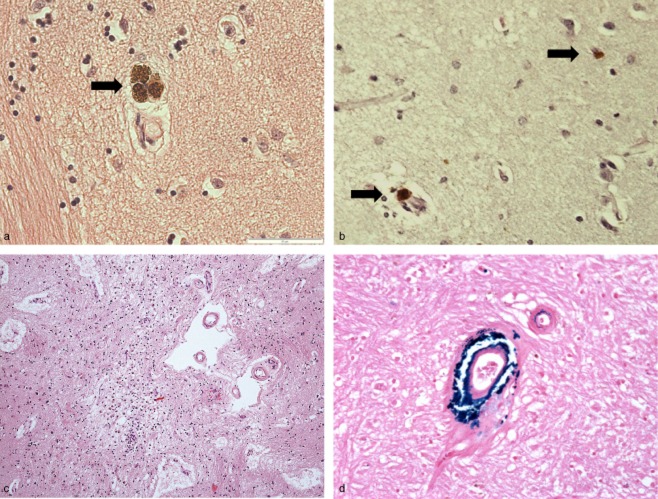
(a,**b**) Haemosiderin deposits. Grouped clusters of several profiles (**a**; arrow) were counted as a single focus. Foci of haemosiderin were identified in both periarterial (and arteriolar) and pericapillary locations (**b**; arrows). (**c**) Perivascular attenuation was interpreted as parenchymal ‘loosening’ and vacuolation around arterioles and small arteries, or within parenchyma, whether or not associated with gliosis. (**d**) Perivascular stainable-iron deposition was confirmed using Perl's staining. (**a**,**b**,**c** haematoxylin and eosin; **d** Perl's stain: **a**,**c** ×40 obj; **b** ×10 obj; **d** ×20 obj.)

Formalin-fixed blocks, processed and embedded in paraffin wax, were sectioned at 6 μm and stained with haematoxylin and eosin (H&E). Histological evaluation of focal haemosiderin deposits were assessed in the putamen at coronal levels corresponding to levels 11–14 of the Newcastle Brain Map (https://nbtr.ncl.ac.uk). In a subgroup of cases the presence of haemosiderin was confirmed using Perls' Prussian blue stain (Figure 1**d**). The total number of discrete perivascular and/or neuropil deposits of haemosiderin (as single profiles or clusters of profiles) in the putamen was counted blind to any clinical or pathological data (Figure 1**a**,**b**). The area of the putamen in each section was measured using a point-counting approach: A clear acetate marked with points in a 2-mm grid pattern was placed over the microscope slide with random orientation and position. The number of points falling over the putamen was counted. This process was repeated five times and the mean of these counts calculated and multiplied by 0.04 to give the cross-sectional area in cm^2^. The density of haemosiderin deposits was expressed for statistical analysis as number per unit area of tissue.

In the same H&E-stained sections the presence of vascular pathology and ischaemic parenchymal damage was assessed and recorded. These markers included the presence of: atheroma of larger perforating arteries; significant arterial and arteriolar sclerosis; microinfarcts; perivascular (Figure 1**c**) or more widespread attenuation and rarefaction of the parenchyma (often associated with neuronal loss and astrogliosis), arteriolar microaneurysm formation.

Other pathological data on the donors were obtained from the archives of the MRC CFAS (http://www.cfas.ac.uk). These included CERAD and Braak scores for Alzheimer plaques and tangles and evaluations of cerebrovascular disease, especially cerebral infarcts, lacunes and SVD. SVD in CFAS is defined as the presence of one or more of the following: moderate or severe arteriosclerosis and/or arteriolosclerosis, microinfarcts, severe white matter attenuation 18,22. CFAS collects information from study respondents and informants including the presence of dementia, stroke, diabetes and heart disease 23.

### Immunohistochemistry and quantification

Deparaffinized 6-μm sections of the putamen were incubated with 3% H_2_O_2_ in methanol for 20 min to block endogenous peroxidase activity. Sections were microwaved in trisodium citrate solution (pH 6.5) for antigen retrieval and blocked with 1.5% normal sera for 30 min before incubation with the primary antibody for 1h at room temperature [glial fibrillary acidic protein: GFAP (1:500, Dako, Ely, UK); CD68 (1:100, Dako); CD163 (1:100, Serotec, Kidlington, UK); fibrinogen (1:400, Alere Ltd, Stockport, UK); ferritin (1:1000, Sigma, Poole, UK)]. The avidin-biotin horseradish peroxidase (ABC-HRP) complex method was used (Vectastain Elite kit, Vector Laboratories, Peterborough, UK), with diaminobenzidine (DAB) as the substrate. Five random regions within the area of interest were selected (×20 magnification; Cell∧R, Olympus, Southend-on-Sea, UK), and the percentage area immunoreactivity of the image analysed using analysis^∧^D software (Olympus Biosystems, Planegg, Germany) following delineation and exclusion of vascular profiles and voids in the sections.

### MRI scanning of the frontal lobe

**Figure 2:**
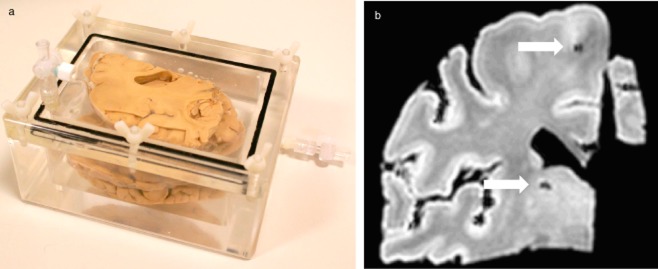
(**a**) Perspex chamber loaded with formalin fixed frontal lobe brain slices used to present tissue for magnetic resonance imaging (MRI). (**b**) Susceptibility weighted 3T MRI scan image of a representative slab of brain tissue showing two signal voids (arrows) with the characteristics of microbleeds.

Magnetic resonance imaging analysis to detect CMB profiles were investigated in 12 cases. These included six cases with the highest frequencies of focal haemosiderin deposits, as assessed by histological examination, compared with six with the lowest burden of focal haemosiderin. For each case five formalin-fixed coronal slices of the frontal lobe (5–8 mm thick) were submerged in fomblin oil (Solvay Solexis, Spinetta Marengo, Italy) in a custom built Perspex chamber (Figure 2**a**; Royal Hallamshire Hospital, Engineering Workshop). MRI (Philips, Eindhoven, the Netherlands) was performed at 3.0Tesla using a susceptibility-weighted protocol optimized for *post mortem* brain imaging. The parameters for the susceptibility weighted sequence were: repetition time 29 ms; echo time 15 ms; flip angle 15°; voxel size 0.45 × 0.45 × 1 mm (slice thickness 1 mm); number of excitations 2; acceleration factor 1.2. For conventional gradient echo T2 weighted sequences the parameters were: repetition time of 500 ms; echo time 16 ms; flip angle 16°; voxel size 0.45 × 0.44 × 2.0 mm (slice thickness 2 mm); number of excitations 3. The number of CMB in the MRI images was scored by consensus blinded to any clinical or neuropathological information (B.M.J./N.H.). CMB were defined as foci of blooming artefact up to 5 mm in diameter that may represent microhaemorrhages taking care to count away from sulci to avoid air/fluid interface artefact. The number of CMB present in each brain scan was counted and adjusted for the size of the tissue slab. This was achieved by dividing the CMB count in each slice by the surface area expressed as the number of pixels in standardized MRI images. As all brain slices were scanned using the same apparatus and scanner the only variation in image size was due to brain size.

### Statistical analysis

Inter-rater reliability for haemosiderin counting was assessed using Spearman Rank correlation, with additional analysis of inter-observer bias (paired *t*-test) and reproducibility (mean and 95% confidence interval of inter-observer difference).The strength of association of focal putaminal haemosiderin deposition and global pathology, local neuropathology, clinical information and molecular markers and the HFE H63D genotype was assessed using either the Wilcoxon Rank Sum Test or the K Sample Median Test. Analyses were performed using STATA version 12.0.

## Results

### Haemosiderin deposition in the putamen in ageing brains

**Figure 3:**
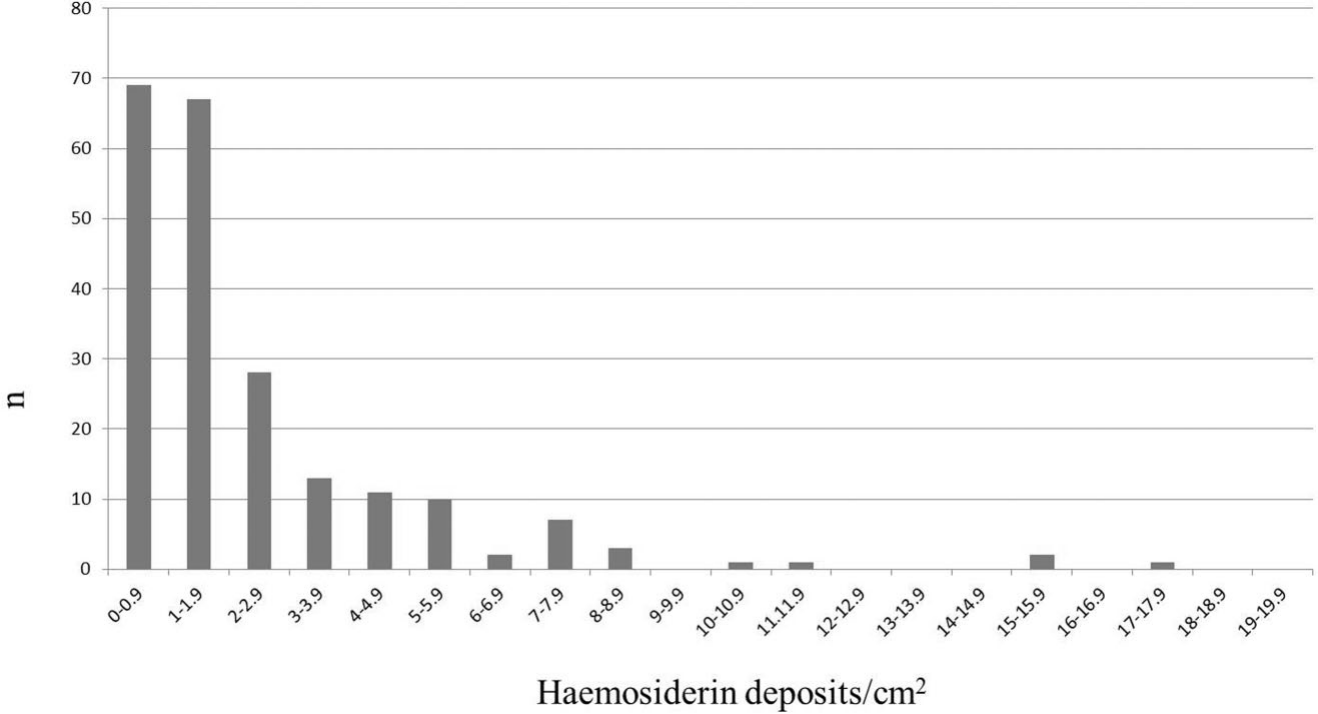
Bar chart showing distribution of haemosiderin density in the putamen across the cohort.

Putaminal haemosiderin deposition, evident as crystalloid profiles varying from dark brown to a lighter reddish-brown granular material, occurred in 99% of the ageing population aged 65 and older (198/200 cases), as assessed in H&E-stained sections (Figure 1**a**,**b**). There was significant association between haemosiderin deposition identified in H&E sections and by the Perls' Prussian blue method (Figure 1**d**; *P* < 0.001; Wilcoxon Rank Sum test). A significantly higher number of haemosiderin deposits were detected in periarterial/periarteriolar regions (mean 7.68 ± 0.952) compared with parenchymal (pericapillary) locations (2.79 ± 0.55) (*P* < 0.001) (Figure 2**b**). There was good agreement between the counts of the two observers (P.G.I., B.M.J.: Spearman: *r* = 0.89, *P* = < 0.001) and there was no evidence of inter-observer bias (t = 1.83, *P* > 0.08; mean inter-observer difference = 20.4, 95% confidence interval −2.8 to 43.61). Figure 3 shows the distribution of focal haemosiderin counts within the cohort. While two-thirds of the cohort have a density below two deposits per cm^2^ there is a large tail of cases with more frequent haemosiderin deposition.

### Relationship of haemosiderin to global neuropathology and local vascular pathology

There was no evidence that haemosiderin deposition in the putamen was related to severity of whole brain measures of neuropathology, including Braak stage (*P* = 0.88), CERAD senile plaque severity (*P* = 0.53) or presence of synucleinopathy (*P* = 0.83), amyloid angiopathy (*P* = 0.36) and SVD (*P* = 0.36). Comparison with whole brain assessment of presence of lacunes showed a positive association with severity of haemosiderin deposits (*P* = 0.023).

**Figure 4:**
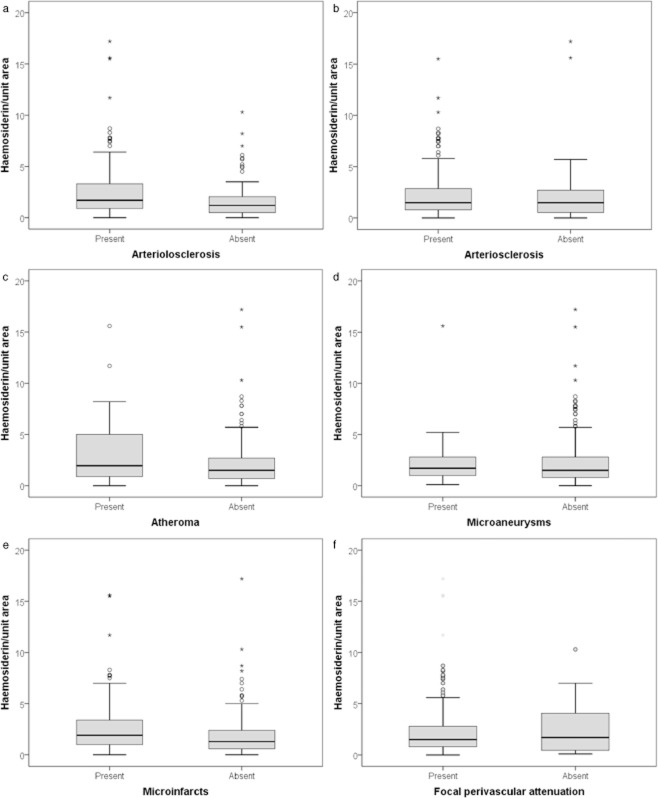
Box and whisker plots showing relationship between the density of haemosiderin deposition and both local (**a**,**c**–**f**) and global (**b**) measures of vascular pathology.

There were significant associations with indices of local vascular pathology, including both pathology of small vessels and ischaemic parenchymal lesions, in the putamen. The intensity of haemosiderin deposition was higher in people with putaminal microinfarcts (*P* = 0.015), arteriolosclerosis (*P* = 0.022) and changes of perivascular attenuation (*P* < 0.001), but no association was found with atheroma (*P* = 0.13), arteriosclerosis (*P* = 0.17) or microaneurysm (*P* = 0.51), as shown in Table 1 and Figure 4.

**Table 1:** Association between putaminal haemosiderin deposition, brain pathology scores, local vascular pathology measures and cerebrovascular risk factor clinical data

		Absent		Present		*P*
		Median	IQR	Median	IQR	
Global pathology	CAA	–	–	–	–	0.77
	Braak stage	–	–	–	–	0.88
	Senile plaque score	–	–	–	–	0.21
	Synucleinopathy	–	–	–	–	0.83
	Small vessel disease	1.6	0.8–2.6	1.7	0.9–3.0	0.36
	Lacunes	1.4	0.7–2.6	2.1	0.9–4.6	0.02
Local pathology	Arterial sclerosis	1.6	0.8–2.8	1.5	0.5–2.6	0.17
	Arteriolosclerosis	1.3	0.9–3.3	1.7	0.6–2.2	0.02
	Atheroma	0.5	0.9–4.6	1.8	0.6–2.7	0.13
	Microinfarcts	1.3	0.9–3.3	1.9	0.6–2.5	0.01
	FPVA	1.1	0.9–3.2	1.8	0.4–1.8	0.0003
Clinical risk factors	Age	–	–	–	–	0.01
	Gender	–	–	–	–	0.41
	Diabetes	–	–	–	–	0.90
	Stroke	–	–	–	–	0.45
	Heart disease	–	–	–	–	0.44
	Dementia	–	–	–	–	0.34

IQR, interquartile range; CAA, cerebral amyloid angiopathy; FPVA, focal perivascular attenuation.

### Relationship of focal haemosiderin to molecular markers of pathology, gliosis and tissue integrity in the putamen

CD68^+^ microglia were predominantly of a highly branched morphology and were evenly distributed throughout the putamen and did not appear associated with haemosiderin deposition (*P* = 0.69). A distinct subset of CD163^+^ perivascular macrophages was detected in some cases and were significantly correlated with haemosiderin deposition (*P* = 0.005). The density of GFAP immunoreactive astrocytes (*P* = 0.261), myelin density (determined by immunostaining for MBP; *P* = 0.35) and ferritin immunoreactive cells (*P* = 0.79), predominantly oligodendrocytes and astrocytes, were not related to haemosiderin deposition.

### Relationship to clinical information and HFE genotype

Analysis of the extent of focal haemosiderin deposition was statistically analysed in relation to data related to brain weight, age and self-reported clinical parameters relevant to cardiovascular and cerebrovascular risk factors. Higher haemosiderin deposition was significantly associated with increasing age (Spearman's Rho = 0.22, *P* = 0.0016) and lower brain weight (*P* < 0.001), but was not associated with brain atrophy (*P* = 0.25), dementia (*P* = 0.34), diabetes (*P* = 0.90), gender (*P* = 0.68), myocardial infarction (*P* = 0.44), stroke (*P* = 0.45) and systemic hypertension (*P* = 0.49).

Previous HFE genotyping of the H63 locus in these individuals showed that 66.1% were homozygous for the wild-type allele (H/H), 30.4% were heterozygous (H/D) and 3.6% homozygous (D/D) 19. Haemosiderin burden was not significantly higher in HFE H63D carriers compared with noncarriers (*P* < 0.053), although the relationship came close to conventional statistical significance. However this component of the study has rather limited power due to the small sample size for a genetic association analysis and needs to be repeated in a larger cohort.

### Correlation of pathological evidence of haemosiderin in the putamen with MRI evidence for microbleeds in other brain areas

Formalin fixed frontal lobe brain tissue coronal slices underwent MRI analysis at 3.0T in a custom built Perspex chamber (Figure 2**a**), and showed profiles corresponding to typical microbleed signal voids (Figure 2**b**). The MRI method was optimized to ensure that the signal voids demonstrated most likely correspond to CMB as described in clinical imaging of living patients 2. The use of an immersion oil was found to minimize artefacts associated with air and water interfaces and edge artefacts. Comparison of the frequency of CMB profiles in six cases selected with high frequency of putamen focal haemosiderin deposition and six cases selected with low deposition showed that more microbleeds (predominantly in a frontal white matter distribution) is shown in Table 2. The area of CMB in MRI images from cases with high putamen haemosiderin counts was significantly increased (*P* = 0.003).

**Table 2:** Comparison of area of MRI CMB in frontal lobe tissue slices in brains characterized by high (6) and low (6) focal haemosiderin counts in the putamen

Area of MRI cerebral microbleeds (CMB pixels/total pixels)					
Category	Total CMB	IQR	25th centile	75th centile	*P*
High putamen haemosiderin	0.697	0.356	0.545	0.89	
Low putamen haemosiderin	0.28	0.226	0.167	0.391	0.003

## Discussion

It is widely assumed that MRI CMB reflect extravasation of red blood cells from cerebral blood vessels, resulting in pericyte erythrophagocytosis, haemoglobin degradation and haemosiderin deposition 1–3,5. In this population-based neuropathology study we report the prevalence of putamen focal haemosiderin deposition assessed by light microscopy and show that it is significantly associated with indices of SVD, age and low brain weight. The *HFE* H63D genotype was not significantly associated with severity of haemosiderin deposits in this cohort. We propose that accumulation of focal haemosiderin deposits in older peoples brains in part reflects the inability of the ageing brain to store ferritin iron released from ischemic damage to oligodendrocytes and other cells because of a reduced overall population of remaining healthy brain cells. Oligodendrocytes are recognized to be vulnerable to ischaemia during development but there is increasing evidence of similar vulnerability in adult white matter diseases 24

Basal ganglia structures contain the highest concentration of iron in the brain 25. Previous histological analysis of the putamen in the ageing population has suggested that haemosiderin deposition primarily occurs at the capillary level 3, in contrast we report a significantly higher number of haemosiderin deposits in periarterial/periarteriolar regions compared with pericapillary locations. This difference may reflect the large sample size, and population-based sampling, of the CFAS cohort investigated in this study, compared with the previous report (33 cases) 3.

Cerebral microbleeds are small MRI signal voids indicative of focal haemosiderin deposition. Several MRI studies have investigated the prevalence of microbleeds in the ageing population, and report CMB frequencies ranging from 3% to 38% 4,26–29. In contrast to these MRI studies, we report histological detection of focal haemosiderin deposition in 99% of CFAS cases aged 65 years and over, suggesting that histology is currently a more sensitive technique for detecting haemosiderin in *post mortem* brain tissue than MRI analysis. MRI parameters for the detection of CMB vary between these studies and likely contribute to the wide range of prevalence reported. For example increasing the magnet strength from 1.5T to 3.0T has been shown to increase the number of detectable of CMB 30. Emerging experience of imaging at higher field strengths suggest a predictable increase in rate of detection, and the apparent size of CMB detected 31. However, in line with these imaging studies, we report a significant positive association between haemosiderin deposition and age 4,26–29. Furthermore, consistent with MR CMB evidence from the Rotterdam Scan Study 4, but not the Age, Gene/Environment Susceptibility (AGES) Reykjavik Study 28 nor the Framingham Study 26, we report no significant association between gender and prevalence of focal haemosiderin deposits. Taken together these data support the hypothesis that haemosiderin deposits need to accumulate to a sufficient size, or ferromagnetic potential, in order to become detectable as MRI lesions. We suggest that there is no pathogenetic or qualitative difference between histological focal haemosiderin and MRI CMB, simply a matter of a ‘size’ threshold.

Increased focal haemosiderin deposition in brains from the CFAS population significantly correlates with indicators of SVD, including microinfarcts, arteriolosclerosis, lacunes and perivascular attenuation, concordant with data from population-based MRI analyses 27,29, but not measures of global neuropathology. In contrast to studies which suggest that the prevalence of CMB impacts cognitive function in stroke clinic patients 32,33 and a population-based ageing cohort 34, we report no significant correlation between focal haemosiderin deposition and dementia status. CMB are simultaneously located in a variety of brain regions, including subcortical white matter and the basal ganglia, in SVD patients 35,36. In the current study, cases with the highest levels of haemosiderin deposition in the putamen also have MRI-detectable CMB in the frontal lobe, predominantly in the white matter, suggesting that CMB may reflect widespread SVD in the ageing brain. MRI-based correlations with dementia status derived from clinical case-control studies are not directly comparable to the present population-based data as they likely select for cases with the high levels of haemosiderin that may be less frequent at a population level.

It is generally assumed that the CMB detected by MRI represent sites of microhaemorrhage which result in extravasation of erythrocytes and give rise to small foci of chronic blood products and haemosiderin deposition. This concept is curious as there is no established literature about similar minor spontaneous extravasations in peripheral tissues lying outside the blood–brain barrier. An evaluation of skin in the leg related to chronic venous stasis for example found perivascular haemosiderin to be infrequent and usually associated with local inflammation 37. The microbleed literature often refers to an older study in which the presence of microaneurysms (of Ross Russell) was related to the presence of small haemorrhages 38. However the ‘small’ haemorrhages described in that paper were detected macroscopically, not by microscopic examination, as lesions ‘less than 30 mm in diameter’ and they were present in patients with severe hypertension (defined as diastolic blood pressure > 110 mmHg accompanied by cardiomegaly). This study group from 1967 is likely to be very different from the present day elderly medicated population, and the type of lesion described is likely to be different from the small foci of haemosiderin detected by microscopy and as MRI CMB in the modern literature. In contrast to this idea of an origin from extravasation we propose that focal haemosiderin deposits may arise from local iron sources within the brain. Iron stored within ferritin, the iron storage protein, is predominantly associated with oligodendrocytes in the CNS 39. Dysregulation of iron homeostasis can result in increased oxidative stress and ultimately neurodegeneration 40, therefore iron content in the CNS is strictly regulated by a number of proteins, including HFE 41. The H63D polymorphism in the *HFE* gene is associated with increased iron uptake and ultimately iron overload 42–46. In this study we were unable to demonstrate a significant association between the *HFE* H63D polymorphism and greater burden of haemosiderin deposition. Most of the H63D mutation detected was heterozygous, in which dysregulated iron uptake is less pronounced, and where increased iron uptake does not give rise to haemochromatosis. The cohort size is also rather small for a genetic association study. A more definitive test of our hypothesis, given the modest power to test it using these genetic data, would be to make direct measurements of brain iron content for comparison with data on CMB and microscopical focal haemosiderin deposits. There is also an urgent need for better histopathological studies to characterize the range and threshold of haemosiderin pathology that can give rise to an MRI microbleed artefact.

Haemosiderin deposition in this study was associated with elevated expression of CD163 immunoreactive perivascular cells, a haem scavenger receptor expressed by macrophages 47,48. The lack of a characterized functional iron export pathway from the brain likely results in the perivascular accumulation of haemosiderin, some of which may be mobilizable via macrophage activity. While our data do not exclude the possibility that this is a response to extravasated erythrocytes we did not observe recent perivascular haemorrhage in any of our cases. The presence of perivascular haemosiderin in CADASIL cases, in which there is massive arteriolar fibrosis, no evidence of a clinical propensity for haemorrhage, and very severe ischaemic white matter degeneration, further supports the possibility that deposited iron can arise from damaged parenchyma rather than being vascular in origin. Of interest the chief neuropsychological correlates associated with CMB are precisely those now invoked as the core features of subcortical ischaemic encephalopathy related to small vessel ischaemia 33,49,50.

It is potentially important to distinguish CMB, and the pathological correlates of haemosiderin deposition, in different anatomical loci. CAA is associated with a high frequency of cortical MRI CMB 1. Histopathology of CAA shows microaneurysm formation, inflammation, small perivascular bleeds and microinfarction 7. Clinically CAA is undoubtedly a major risk factor for lobar haemorrhage. We therefore suggest that CMB in a cortical distribution likely do have a role as a biomarker for risk of clinical haemorrhage due to underlying CAA in older people at risk of that pathology. Our data, in contrast, are consistent with the hypothesis that white matter and basal ganglia focal haemosiderin/CMB deposits are frequently ischaemic in origin and have different biomarker implications. In terms of the predictions addressed in this study we have demonstrated that focal haemosiderin deposition is significantly associated with, predominantly local, indices of ischaemic SVD but not to neurodegeneration, large vessel disease and vascular pathology in other brain regions, and that people with a higher burden of focal haemosiderin deposits (and small vessel ischaemia) in the putamen have more CMB in other brain areas. Further clinical and pathological studies are needed to address this ischaemic hypothesis for the origin of CMB.
